# Cross-cultural validation of the Short-Form Vascular Access Questionnaire

**DOI:** 10.1177/11297298231170407

**Published:** 2023-06-02

**Authors:** João Pedro Barros, João A. Fonseca, Rui Pinto, Jorge Pratas, Ricardo Cruz-Correia

**Affiliations:** 1 Programme in Health Data Science, Faculty of Medicine, University of Porto, Porto, Portugal; 2 NephroCare, Portugal; 3 CINTESIS@RiSE, MEDCIDS, Faculty of Medicine, University of Porto, Porto, Portugal; 4 Centro Hospitalar e Universitário de Coimbra, Coimbra, Portugal; 5 Department of Community Medicine, Information and Decision in Health, Faculty of Medicine, University of Porto, Porto, Portugal; 6 Center for Health Technology and Services Research, Faculty of Medicine, University of Porto, Porto, Portugal

**Keywords:** End stage renal disease, renal dialysis, cross-cultural validation, vascular access, patient reported outcomes measure

## Abstract

**Background:**

Vascular access (VA) is a central condition for hemodialysis (HD). Screening patients’ views regarding their VA is a significant end point for improving the quality of care. The Short-form Vascular Access Questionnaire (SF-VAQ) is a specific questionnaire to assess patients’ satisfaction levels regarding their VA.

**Purpose:**

This study aims to develop the Portuguese version of the SF-VAQ and assess its psychometric properties.

**Methods:**

A forward and back translation was used. A multicentric study was conducted with 156 patients undergoing hemodialysis to psychometric testing. Reliability (internal consistency and test-retest) was assessed using Cronbach’s alpha and Intraclass Correlation Coefficient. A construct validity test was conducted using factor analysis. The convergent validity was calculated using the correlation coefficient.

**Results:**

An obtained Cronbach’s alpha of 0.77 indicates good internal consistency. The test-retest reliability was established using the Intraclass Correlation Coefficient (ICC) of 0.771. The four sub-scales proposed by the instrument’s designer were confirmed, which together accounted for 53% of the variance. The correlation with the Visual Analogue Scale was *r* = 0.895 (*p* < 0.001), confirming the convergent validity.

**Conclusion:**

The Portuguese version of the SF-VAQ is a valid and reliable instrument with good psychometric properties to be implemented to promote an evaluation of VA satisfaction in HD patients and improve patient care.

## Introduction

Patients diagnosed with Chronic Kidney Failure (CKD) performing Hemodialysis (HD) as a Renal Replacement Therapy (RRT) need functioning Vascular Access (VA).^1^ Arteriovenous Fistula (AVF) is internationally recognised as the optimal VA for HD patients because it is associated with lower morbidity and mortality than central venous catheters (CVC) and arteriovenous grafts (AVG).^2^ Prior VA guidelines suggest ‘fistula first’ as a priority.^3^ Patients with AVF have higher Health-Related Quality of Life when compared with AVG and CVC.^4^ However, AVF is associated with multiple radiological interventions and complications due to cannulation sites and other nursing practices, which can influence and reduce a patient’s Quality of Life (QoL). Taking into account a patient-centred approach, this concept evolved to a new personalised approach regarding a VA choice: ‘the right access, to the right patient, at the right time, for the right reasons’.^2,3^

Modern^2,5^ healthcare teams aim to reduce symptoms, improve patient’s condition and assess how patients feel. The patient reports with clinical outcomes, such as QoL, survival, or permanent loss of VA, play an increasing role in clinical judgement.^2,6^ These Patient Reported Outcome Measures (PROMs) are self-reports and standardised instruments used to appraise outcomes related to QoL, well-being, symptoms, functional status and other psychological and social aspects.^7^ The outcomes achieved through PROMs have a critical preponderance in the decision-making process and have been adopted as a cornerstone for patient-centred care.^2,6–8^

Conducted studies with the implementation of PROMs assessing VA in HD accomplish quality improvement programs, cultivating quality in care, minimising and evaluating the impact in QoL as a priority for all HD stakeholders.^6,9^

Due to the lack of instruments and tools available in Portuguese for assessing patients’ satisfaction with VA, the Short-Form -Vascular Access Questionanaire (SF-VAQ) was chosen for adaptation. This tool is well-established and easy to administer to patients.^10^

The SF-VAQ assess patient satisfaction regarding their VA in four domains: Overall Satisfaction (one item), Physical Domain (four items), Social Domain (four items) and Dialysis Complications (four items). The item has a 7-point Likert scale that ranges from 1, where Strongly Disagree, to 7, where Strongly Agree. The score in the Physical, Social and Dialysis Complication domains can be 28 points each (four questions multiple by 7 Likert point scales). The total score of these domains can have 84 points.^10^

This study aims to conduct the translation and cross-cultural adaptation and validation of the SF-VAQ for Portuguese. As secondary objectives, this study seeks to assess the satisfaction levels regarding the VA type, characterise sample sociodemographic VA characteristics and their impact on the VA satisfaction levels.

## Methods

### Study design

A prospective methodological study with psychometric evaluation was conducted in three stages: (1) the translation process of the original scale, (2) a field test of the translated instrument and (3) the primary analysis assessing the reliability (internal consistency and test-retest), structural validity and convergent validity of the SF-VAQ.

The Beaton et al.^11^ methodology was adopted for translation procedures. A five-stage method includes:

Stage 1 Two translators with different profiles and backgrounds; one did not have any medical or clinical knowledge.
Stage 2 A common translation (T-12) is obtained, and issues related to translation procedures are solved.
Stage 3 Two blind translators who receive the back-translation must translate the questionnaire back into the original language. The mother language of the two back translations (BT-1, BT-2) is the source language of the questionnaire, English.
Stage 4 This stage requires a committee composed of health and language professionals to achieve a cross-cultural equivalence.
Stage 5 In stage 5, a pretest was performed with a minimum of 10 patients to infer the scale’s interpretation.^11^

The study is multicentric, with a sample recruited from two dialysis centres. The inclusion criteria were to be over 18 years old and have been in HD treatments for over 3 months. The study period was a month, and data were collected twice, 4 weeks apart. The capacity of the participants to answer the questionnaire was assessed by the six-item screening method for cognitive impairment.^8^

The sample characteristics regarding the main study variables were analysed using percentages, mean, median and standard deviation (SD). Also, the sample Charlson Comorbidity Index, which records the mortality risk at categorising patients’ comorbidities based on the International Classification of Diseases (ICD), and the Barthel index assesses functional independence, was used for the sample description.

The SF-VAQ results were described using mean and SD and calculated for each domain of the scale and the different VA types. Across the scale, fields were analysed for the obtained results for each VA type. A multivariable analysis was conducted to assess the performed to identify independent predictors using the sociodemographic factors and current VA factors reported.

The scale’s reliability was calculated using the internal consistency (Cronbach’s alpha) and test-retest reliability by Intraclass Correlation Coefficient (ICC) and Weighted Kappa.

The structural validity was assessed by conducting an Exploratory Factor Analysis (EFA). The number of factors to extract was calculated as a minimum eigenvalue of 1 and determined by parallel analysis. The cut-off for factors loading was established as ⩾0.40.^12^ Oblique Promax rotation procedures were used as the factor rotation method because scale factors were assumed to be correlated.

To assess structural validation and convergent validity tested by Pearson’s *r* correlation with a Visual Analogue Scale (VAS) and Bland-Altman test. A Bland-Altman plot was created, with the calculation of the limits of agreement, which implies that 95% of the differences will lie between the upper and lower limits^13^: The cut-off value for significance in this study was set as *p* < 0.05.

LoA9514meandifference±1,96×difference

Also, a Bland-Altman plot determines the reliability of the measurement tool. The differences between the agreements against their mean can represent 95% of the agreement limits.

The ICC was calculated, and applied a *t*-test to verify a systematic bias between the two tests. The following formula was used to calculate the Standard Error of Measurement (SEM) and the Minimal Detectable Change (MDC)^13^:

$$\mathrm{MDC}95=1.96\times \sqrt{2}\times \mathrm{SEM}$$

$$\mathrm{SEM}=\mathrm{SD}\times \sqrt{1}-\mathrm{ICC}$$

### Ethical approval

The study obtained the Ethical Committee appraisal on (06/09/2021) for implementation in two dialysis centres, and patients recruited for the study gave their written consent to participate.

### Data analysis

The collected data were analysed using IBM SPSS (IBM Corp., Armonk, NY) and Rstudio software with the ‘psych’ package.^14^

## Results

### SF-VAQ

The final Portuguese version of the SF-VAQ was obtained after a pretest with 10 patients. Some wording change was conducted for the questions regarding the VA taking care in the Social Domain, leading to the final version and improving the scale interpretation.

## Sample description

A potential sample of 168 hemodialysis patients who visited the two dialysis units met the inclusion criteria. Twelve patients did not complete the questionnaire accurately. The total number of participants in the study was 156 HD patients who were interviewed in the dialysis centre, with a response rate of 93%. The mean age was 70.33 (±12.8) years, 101 were males (64.7%), 116 were married (74.3%) and 129 were retired (82.6%). The vintage dialysis mean was 59.8 (± 68.2) months, and participants had a high level of comorbidities with a Charlson Comorbidity Index mean of 3.87(±0.5) (Table 1).

**Table 1.** Socio-demographic sample characteristics and description of vascular access.

Sociodemographic characteristics		
Age years (mean/SD)	70.33 (±12.8)	
Gender *n* (%)		
Male	101 (64.7%)	
Female	55 (35.3%)	
Marital status *n* (%)		
Married	116 (74.3%)	
Divorced	11 (7.0%)	
Single	12 (7.7%)	
Widowed	17 (11.0%)	
Educational status *n* (%)		
Primary school	136 (87.2%)	
High school	15 (9.6%)	
University	5 (3.2%)	
Work status *n* (%)		
Woking	21 (13.5%)	
Retired	129 (82.6%)	
Housewife	6 (3.9%)	
Hemodialysis vintage months (median)	37.5	
Comorbid conditions *n* (%)		
One	99 (63.5%)	
Two or more	57 (36.5%)	
Diabetes *n* (%)		
Yes	55 (35.3%)	
No	101 (64.7%)	
BMI (mean/SD)	27.1(±4.6)	
Charlson Comorbidity Index (mean/SD)	3.87(±0.5)	
Barthel score mean SD (mean/SD)	91.87(±19.4)	
ESRD Cause (ICD-10) *n* (%)		
E10.2 Type 1 diabetes mellitus with kidney complications	19 (12.2%)	
E11.2 Type 2 diabetes mellitus with kidney complications	11 (7.0%)	
E14.2 Unspecified diabetes mellitus with renal complications	7 (4.5%)	
N18.5 Chronic kidney disease, stage 5	10 (6.4%)	
N18.9 Chronic kidney disease, unspecified	42 (26.9%)	
I12.0 Hypertensive chronic disease	9 (5.8%)	
Others	58 (37.2%)	
Vascular access description		
Current AVF/AVG anatomic position (*n*/%) (*n* = 138)	AVF	AVG
Lower arm	28 (20.3%)	0
Upper arm	101 (73.2%)	9 (6.5%)
Upper leg	0	0
Current CVC anatomic position (*n*/%) (*n* = 18)		
Jugular	18 (100%)	
Femoral	0 (0%)	
Other	0 (0%)	
AVF/AVG on dominant arm (*n*/%) (*n* = 138)		
Yes	35 (25.4%)	
No	103 (74.6%)	
Previous CVC (*n*/%) (*n* = 156)		
Yes	82 (52.6%)	
No	74 (47.4%)	
VA previous failure (n/%) (*n* = 156)	32 (20.5%)	
Yes	124 (79.5%)	
No		
AVF/AVG previous intervention (*n*/%) (*n* = 138)		
Angioplasty	72 (52.2%)	
Angiography	41 (29.7%)	
Without intervention	25 (18.1%)	

SD: standard deviation; AVF: arteriovenous fistula; AVG: arteriovenous graft; CVC: central venous catheter.

The most present VA was an AVF in 129 (82.7%) of the participants, followed by CVC in 18 (11.5%) and AVG in 9 (5.8%). The most used anatomic position in AVF and AVG is the upper arm 101 (73.2%), and for CVC Jugular vein 18 (100%). More than half of the sample had the VA in the non-dominant arm 103 (74.6%). Most of the patients have a previous use of CVC 82 (52.6%) and 32 (20.5%) have a prior failure of the VA. The sample had a high incidence of radiological intervention at the current VA, with 72 (52.2%) submitted to angioplasty and 41 (29.7%) to angiography (Table 1).

## SF-VAQ results

Table 2 summarises the SF-VAQ results. The patients answer the SF-VAQ without assistance and take 10 min to complete the survey.

**Table 2.** SF-VAQ results and domains mean and sum scores.

Domain	Question	Metrics		*N*	Overall		*N*	AVF		*N*	AVG		*N*	CVC		*p* Value
					Mean	SD±		Mean	SD±		Mean	SD±		Mean	SD±	
Overall satisfaction with vascular access	I am satisfied with my vascular access			156	5.33	1.35	129	5.44	1.25	9	4.89	1.45	18	4.72	1.77	0.163
	During the past 4 weeks I was bothered by pain associated with my vascular access	Mean	Overall – 7.75	156	2.80	2.09	129	2.94	2.12	9	4.00	1.87	18	1.22	0.94	<0.001**
			AVF – 8.08													
			AVG – 10.11													
			CVC – 4.22													
	During the past 4 weeks I was bothered by bleeding with my vascular access	Median	Overall – 7	156	1.57	1.44	129	1.63	1.50	9	1.89	1.76	18	1.00	0.00	0.147
			AVF – 7													
			AVG – 9													
			CVC – 4													
Physical function	During the past 4 weeks I was bothered by swelling associated with my vascular access	First quartile	Overall – 4	156	1.71	1.48	129	1.79	1.56	9	1.89	1.53	18	1.00	0.00	0.051
			AVF – 4													
			AVG – 6													
			CVC – 4													
	During the past 4 weeks I was bothered by bruising associated with my vascular access	Third quartile	Overall – 10	156	1.68	1.53	129	1.73	1.55	9	2.33	2.39	18	1.00	0.00	0.056
			AVF – 10													
			AVG – 14.5													
			CVC – 4													
	During the past 4 weeks my access interfered with my daily activities (e.g. work, social, leisure activities or other regular daily activities)	Mean	Overall – 9,09	156	2.81	2.19	129	2.78	2.21	9	3.56	2.18	18	2.72	2.08	0.571
			AVF – 8.51													
			AVG – 10.33													
			CVC – 12.66													
Social function	During the past 4 weeks I was bothered by the appearance of my vascular access	Median	Overall – 7	156	2.96	2.33	129	2.70	2.24	9	3.89	2.71	18	4.33	2.27	0.017*
			AVF – 6													
			AVG – 11													
			CVC – 13													
	During the past 4 weeks my access interfered with my sleep	First quartile	Overall – 4	156	1.59	1.57	129	1.50	1.44	9	1.67	2.00	18	2.22	2.15	0.199
			AVF – 4													
			AVG – 5.5													
			CVC – 5.75													
	During the past 4 weeks my access caused me problems when bathing or showing	Third quartile	Overall – 13	156	1.74	1.48	129	1.54	1.28	9	1.22	0.66	18	3.39	2.03	<0.001**
			AVF – 12													
			AVG – 12.5													
			CVC – 18													
	During the past 4 weeks my vascular access had problems (i.e. didn’t work properly)	Mean	Overall – 9.54	156	2.02	1.29	129	1.98	1.31	9	2.11	1.05	18	2.22	1.30	0.489
			AVF – 9.20													
			AVG – 11													
			CVC – 11.22													
	During the past 4 weeks my vascular access was difficult to care for (i.e. dressings, trying to keep access clean and protected)	Median	Overall – 8	156	2.17	1.35	129	2.08	1.35	9	2.56	0.72	18	2.67	1.53	0.063
			AVF – 8													
			AVG – 11													
			CVC – 11.22													
Dialysis complications	During the past 4 weeks I was worried about being hospitalised because of problems with my access	First quartile	Overall – 5	156	2.48	1.71	129	2.40	1.73	9	2.89	1.61	18	2.89	1.60	0.045*
			AVF – 4													
			AVG – 8.5													
			CVC – 6													
	During the past 4 weeks I was worried about how long my vascular access will last	Third quartile	Overall – 12	156	2.87	1.82	129	2.75	1.83	9	3.44	1.50	18	3.44	1.85	0.055
			AVF – 12													
			AVG – 12													
			CVC – 14													

The physical function, social functioning and dialysis complication are out of a possible 28 points from the four questions of each domain multiplied by 7 points Likert scale. Bold values are statistically significant *p* < 0.05; *p < 0.05; **p < 0.001.

The satisfaction domain with VA was high, with a mean score of 5.33 (±1.35). The AVF, AVG and CVC scores were 5.44 ± 1.25, 4.89 ± 1.45 and 4.72 ± 1.77 (*p* = 0.163).

In the Physical function domain, AVG reports higher pain levels (4.00 ± 1.87, *p* < 0.001). The mean sum scores for this domain across the four items were 4.22, 8.08 and 10.11 for CVC, AVF and AVG, respectively, in possible 28 points (four questions Likert 7 points each) (Table 2).

In the Social function, higher Likert scores obtained for CVC and AVG follow AVF regarding physical appearance (4.33 ± 2.27 vs 3.89 ± 2.71vs 2.70 ± 2.24; *p* = 0.017). Also, bathing difficulties present higher values for patients with CVC than AVF and AVG (3.39 ± 2.03). The mean sum scores for these domains across the four items were 8.51, 10.33 and 12.66 for AVF, AVG and CVC, respectively, in possible 28 points (four questions Likert 7 points each) (Table 2).

In the Dialysis Complication domain, concerns about hospitalisation because of the VA are more significant for AVG and CVC than AVF (2.89 ± 1.60 vs 2.89 ± 1.61 vs 2.40 ± 1.73; *p* = 0.045). Patients with CVC and AVG have more concerns about VA longevity, with higher scores when compared with AVF, respectively (3.44 ± 1.85 vs 3.44 ± 1.50 vs 2.75 ± 1.83). The mean sum scores for these domains across the four items were 9.20, 11 and 11.22 for AVF, AVG and CVC, respectively, in possible 28 points (four questions Likert 7 points each) (Table 2).

Elderly patients above 70 years old seem to be more satisfied with the current VA but also with fewer concerns related to body image impact caused by the VA, with a mean response of 5.33 (*p* = 0.027) and 3.38 (*p* = 0.034), respectively (Table 3).

**Table 3.** Sociodemographic and current vascular access characteristics.

Variable	Mean score of the SF-VAQ related to the question:												
	3	4	5	6	7	8	9	10	11	12	13	14	15
Age	*p* = 0.027*	*p* = 0.969	*p* = 0.185	*p* = 0.199	*p* = 0.796	*p* = 0.801	*p* = 0.034*	*p* = 0.318	*p* = 0.770	*p* = 0.996	*p* = 0.859	*p* = 0.212	*p* = 0.779
<60	4.88	2.82	1.39	1.64	1.52	2.55	2.64	1.64	1.64	1.94	2.06	1.94	2.64
60–70	5.78	2.69	1.25	1.28	1.59	2.75	2.06	1.37	1.81	2.19	2.22	2.81	3.06
>70	5.33	2.84	1.75	1.88	1.77	2.93	3.38	1.65	1.75	1.99	2.20	2.56	2.89
Gender	*p* = 0.167	*p* = 0.832	*p* = 0.702	*p* = 0.397	*p* = 0.702	*p* = 0.571	*p* = 0.403	*p* = 0.797	*p* = 0.006*	*p* = 0.108	*p* = 0.035*	*p* = 0.120	*p* = 0.166
Male	5.28	2.86	1.45	1.75	1.64	2.70	2.82	1.51	1.48	1.85	1.96	2.25	2.65
Female	5.42	2.69	1.80	1.62	1.75	3.02	3.20	1.73	2.22	2.33	2.56	2.91	3.27
Previous CVC	*p* = 0.457	*p* = 0.021*	*p* = 0.950	*p* = 0.969	*p* = 0.363	*p* = 0.237	*p* = 0.752	*p* = 0.669	*p* = 0.423	*p* = 0.264	*p* = 0.696	*p* = 0.344	*p* = 0.703
Yes	5.41	3.18	1.56	1.74	1.54	2.98	2.98	1.61	1.82	2.04	2.12	2.51	2.83
No	5.27	2.40	1.59	1.67	1.85	2.60	2.88	1.49	1.62	1.99	2.22	2.44	2.90
Previous VA failure	*p* = 0.732	*p* = 0.933	*p* = 0.191	*p* = 0.656	*p* = 0.668	*p* = 0.710	*p* = 0.128	*p* = 0.424	*p* = 0.050	*p* = 0.032*	*p* < 0.001**	*p* = 0.004*	*p* = 0.010*
Yes	5.38	2.86	1.86	1.90	1.67	2.95	3.50	1.48	1.95	2.60	2.88	3.45	3.67
No	5.34	2.80	1.47	1.64	1.69	2.74	2.72	1.58	1.64	1.80	1.90	2.12	2.57
Current vascular access	*p* = 0.163	*p* < 0.001**	*p* = 0.147	*p* = 0.051	*p* = 0.056	*p* = 0.571	*p* = 0.017*	*p* = 0.199	*p* < 0.001**	*p* = 0.489	*p* = 0.063	*p* = 0.045*	*p* = 0.055
AVF	5.44	2.94	1.63	1.79	1.73	2.78	2.70	1.50	1.54	1.98	2.08	2.40	2.75
AVG	4.89	4.00	1.89	1.89	2.33	3.56	3.89	1.67	1.22	2.11	2.56	2.89	3.44
CVC	4.72	1.22	1.00	1.00	1.00	2.72	4.33	2.22	3.39	2.22	2.67	2.89	3.44
Current AVF/AVG (*n* = 138)	*p* = 0.290	*p* = 0.606	*p* = 0.889	*p* = 0.917	*p* = 0.860	*p* = 0.259	*p* = 0.571	*p* = 0.455	*p* = 0.631	*p* = 0.305	*p* = 0.384	*p* = 0.119	*p* = 0.278
Radiocephalic	5.69	3.10	1.55	1.90	1.63	3.22	3.04	1.73	1.73	1.98	2.12	2.57	2.88
Brachiocephalic	5.33	2.78	1.63	1.76	1.74	2.67	2.76	1.31	1.35	2.22	2.28	2.54	2.89
Brachiobasilic	5.07	3.11	1.85	1.67	2.00	2.37	2.22	1.59	1.52	1.56	1.70	1.81	2.26
Brachioaxillary	5.17	3.83	1.67	1.83	2.17	3.00	3.17	1.00	1.33	2.00	2.33	3.00	3.67
AV dominant arm (*n* = 138)	*p* = 0.142	*p* = 0.565	*p* = 0.677	*p* = 0.956	*p* = 0.509	*p* = 0.146	*p* = 0.538	*p* = 0.330	*p* = 0.997	*p* = 0.089	*p* = 0.074	*p* = 0.072	*p* = 0.028*
Yes	5.07	2.86	1.57	1.82	1.54	2.29	2.64	1.64	1.57	1.61	1.68	1.89	2.21
No	5.49	3.05	1.66	1.79	1.83	2.96	2.81	1.47	1.51	2.09	2.22	2.56	2.95

*p*-Values are from Mann-Whitney or Kruskal-Wallis test, and bold values are statistically significant *p* < 0.05; *p < 0.05; **p < 0.001.

The results from concerns caused by the VA related to baths (*p* = 0.006) and the need to keep the VA clean (*p* = 0.035) have higher scores for females than males (Table 3).

Patient’s with previous CVC-related higher Likert scores for pain (*p* = 0.021). The patients that had a previous CVC reported higher Likert scores about concerns with problems with VA use during HD but also higher values for difficulties related to baths and keeping the VA clean and matters connected with hospitalisation possibility because of the VA, and longevity of the VA (*p* = 0.032; *p* < 0.001; *p* = 0.04; *p* = 0.010) (Table 3).

In the current VA in use, patients report higher pain related to AVG (*p* < 0.001) and higher bothering levels associated with body image-related VA for CVC and AVG, with a mean of 4.33 versus 3.89 (*p* = 0.017). Bathing concerns are more severe for CVC (*p* < 0.001) and higher report values for concerns about hospitalisation because of the VA for CVC and AVG (*p* = 0.045) (Table 3).

The anatomic position of the current VA for AVF and AVG has higher scores for overall satisfaction for radiocephalic, but no statistical differences were found (Table 3).

The VA in the dominant arm influences the report values of VA longevity, with lower scores reported by patients with VA in the non-dominant arm, 2.21 versus 2.95 (*p* = 0.018) (Table 3).

A multivariable analysis was conducted, assuming the score of the SF-VAQ was calculated as a dichotomic variable, assuming values of the upper quartile (*n* = 37, 23.7%). The model identifies younger patients with age below 60 years old (OR: 4.789, 95% CI: 1.161–19.765), female patients (OR: 3.857, 95% CI: 1.467–10.141) and patients without previous VA failure (OR: 2.935, 95% CI: 1.062–8.111) to be the strongest independent predictors of a worse SF-VAQ score.

## Reliability of the SF-VAQ

### Internal consistency

The Cronbach’s alpha was 0.77 for the full scale, and for the different dimensions of the scale, Physical Function was 0.616, Social Function was 0.736 and Dialysis Complications was 0.932.

### Test-retest reliability

ICC were 0.771 for the full scale ranging between 0.724 and 0.957 for the different dimensions. The weighted kaaappa values ranged from 0.51 to 0.83 (Table 4).

**Table 4.** Test-retest reliability results of the Intraclass Correlation Coefficient (ICC), Cronbach’s alpha and weighted kappa.

Question	ICC	Cronbach’s alpha	Weighted Kappa
I am satisfied with my vascular access	0.841 (0.788–0.882)	0.91	0.83
During the past 4 weeks I was bothered by pain associated with my vascular access	0.869 (0.824–0.903)	0.93	0.77
During the past 4 weeks I was bothered by bleeding with my vascular access	0.806 (0.744–0.855)	0.89	0.48
During the past 4 weeks I was bothered by swelling associated with my vascular access	0.737 (0.656–0.801)	0.85	0.66
During the past 4 weeks I was bothered by bruising associated with my vascular access	0.944 (0.924–0.959)	0.97	0.74
During the past 4 weeks my access interfered with my daily activities (e.g. work, social, leisure activities or other regular daily activities)	0.886 (0.847–0.915)	0.94	0.64
During the past 4 weeks I was bothered by the appearance of my vascular access	0.957 (0.941–0.968)	0.98	0.73
During the past 4 weeks my access interfered with my sleep	0.937 (0.915–0.954)	0.97	0.51
During the past 4 weeks my access caused me problems when bathing or showing	0.855 (0.806–0.892)	0.92	0.56
During the past 4 weeks my vascular access had problems (i.e. didn’t work properly)	0.724 (0.64–0.791)	0.84	0.58
During the past 4 weeks my vascular access was difficult to care for (i.e. dressings, trying to keep access clean and protected)	0.769 (0.696–0.826)	0.87	0.76
During the past 4 weeks I was worried about being hospitalised because of problems with my access	0.927 (0.901–0.946)	0.96	0.77
During the past 4 weeks I was worried about how long my vascular access will last	0.832 (0.776–0.875)	0.91	0.73

## Structural validity

An EFA was conducted to assess the tool’s measurement characteristics and the variable’s relationship degree. The suitable data for the factoring analysis was calculated using Bartlett’s test. The results obtained for the KMO of 0.74, χ^2^ = 420.5064 and a *p*-value <0.001 indicate that an EFA can be conducted.^12^

The number of obtained factors is 4, and factor loading range from 0.475 to 0.931. The total variance explained is 53.4% (Table 5).

**Table 5.** Exploratory factor analysis of the SF-VAQ (*n* = 156).

Items	Factors loading			
	Factor 1	Factor 2	Factor 3	Factor 4
3	0.494			
4		0.523		
5		0.525		
6		0568		
7		0.642		
8			0.696	
9			0.931	
10			0.492	
11			0.475	
12				0.917
13				0.909
14				0.856
15				0.858
				
Proportional variance	0.247	0.146	0.102	0.039
Cumulative variance	0.0247	0.392	0.495	0.534

## Convergent validity

The convergent validity of the Portuguese version of the SF-VAQ scale was assessed using a VAS as a comparator. No previous study was found to evaluate the scale convergent validity due to the lack of research studies conducted in this field to use as an external comparator. The obtained results were correlated between each scale and the domains of the scale. The total score of the SF-VAQ and the VAS is *r* = 0.895 (*p* < 0.001) (Table 6). The correlation coefficients with the different dimensions ranged from *r* = 0.335 to *r* = 0.907, with questions 10, 11 and 12 with scores below 0.6

**Table 6.** Convergent Validity of the SF-VAQ.

SF-VAQ	VAS	VAS score	SF-VAQ/VAS dimension
3	0.633**	0.206**	0.633**
4	0.857**	0.364**	0.865**
5	0.554**	0.414**	
6	0.874**	0.482**	
7	0.765**	0.239**	
8	0.849**	0.607**	0.848**
9	0.875**	0.645**	
10	0.486**	0.381**	
11	0.335**	0.339**	
12	0.477**	0.568**	0.888**
13	0.907**	0.578**	
14	0.632**	0.559**	
15	0.851**	0590**	

Pearson’s *r* correlation between the SF-VAQ and the Visual Analogue Scale and Visual Analogue Scale Score;**p < 0.001.

**Figure 1.**
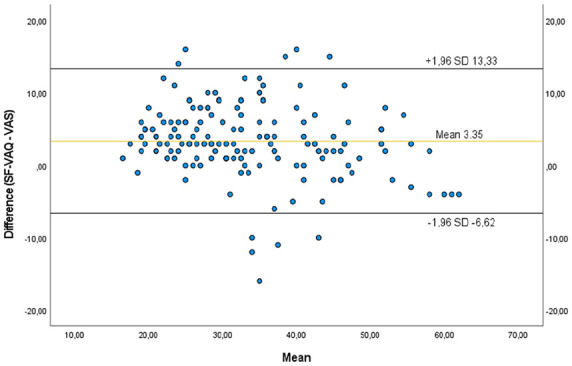
Bland-Altman plot for the mean differences between the SF-VAQ and the VAS. The mean difference is indicated by the solid horizontal line, and the limits of agreement are demarcated by the dashed horizontal lines.

The obtained MDC was 4.65 for the overall score of the scale (Table 7). The obtained plot is presented in Figure 1 with a mean of 3.55 and limits of agreement of 13.33 and −6.62 (Figure 1).

**Table 7.** Standard error of measurement (SEM) and minimal detectable change (MDC) calculation with the SF-VAQ and the VAS score.

Domain	N	SF-VAQ	VAS	Difference mean ± SD	ICC (95% CI)	SEM	MDC
Score	156	31.72 ± 11.42	35.07 ± 10.31	3.35 ± 5.09	0.890 (0.853–0.919)	1.68	4.65
Overall satisfaction	156	5.33 ± 1.35	5.25 ± 1.58	0.07 ± 1.27	0.625 (0.519–0.712)	0.77	2.13
Physical function	156	7.75 ± 4.53	9.23 ± 4.88	1.48 ± 2.34	0.865 (0.819–0.900)	0.85	2.35
Social function	156	9.09 ± 5.77	10.39 ± 4.88	1.30 ± 3.05	0.837 (0.783–0.878)	0.52	1.44
Dialysis complication	156	9.54 ± 5.71	10.19 ± 5.19	0.64 ± 2.62	0.884 (0.845–0.914)	0.21	0.58

## Discussion

This study reports on developing and assessing the Portuguese version of the SF-VAQ to evaluate the patient’s satisfaction level with their VA. To our knowledge, this is the first cross-cultural validation of the SF-VAQ.

The scale shows good internal consistency, with Cronbach’s alpha of 0.77, which compares with 0.84 of the original study of the SF-VAQ.^10^ For the different sales dimensions, obtained values ranging from Physical Function, Social Function and Dialysis Complications were 0.616, 0.736 and 0.932. Matters of at least 0.70 are considered adequate internal consistency.^15^ However, the value obtained for the Social Function can indicate a poor inter-relatedness between items, but considering the number of items presented in this domain can justify the obtained values.^12^

Test-retest reliability is the consistency of responses across repeated questionnaire administrations.^16^ The SF-VAQ was applied two different times, 4 weeks apart, a duration long enough to allow memory effects to fade and not affect the estimated values for test-retest assessment.^12^ The ICC obtained of 0.771 indicate moderate test-retest reliability,^17^ somewhat lower than other studies’ results of 0.92.^10^ The weighted kappa values range from 0.51 to 0.83 and can be considered from moderate (0.41–0.60) to almost perfect (0.81–1.00).^18^ The scale development research did not calculate the weighted kappa, and non-previous studies were conducted assessing this property.

Convergent validity was assessed with Pearson *r* correlation and Bland and Altman limits of agreement plot. The obtained *r* = 0.895 (*p* < 0.001) confirms the convergent validity. The Bland-Altman plot and limits of the agreement confirmed the convergent validity with a mean value of 3.35. The MDC considered the minimal amount of change that is not likely to be due to chance variation in measurement was 4.65 95% CI.

An EFA assess structural validity. The obtained solution of four factors is the same result of the scale development study,^10^ which confirms the scale structural validity and the existence of four different domains.

The study sample’s sociodemographic characteristics were similar to other studies assessing patient satisfaction with the same mean age and AVF as the most frequent permanent VA.^9,10^

The obtained results for satisfaction levels regarding VA obtained higher values for AVF when compared to AVG and CVC, similar to other conducted studies results with AVF received higher scores. AVF and AVG reported higher pain levels, and CVC reported higher physical appearance impact, with the literature studies obtaining similar results.^9,10,19^

The age also influenced the score obtained, with elderly patients above 70 more satisfied with their VA mean of 5.33 and fewer concerns related to the VA appearance mean of 3.38. This fact was also identified with OR: 4.789, 95% CI: 1.161–19.765 for patients below 60 years old. Female patients are more concerned about keeping the VA clean and obtained as OR: 3.857, 95% CI: 1.467–10.141. This result was also accepted as a conclusion in the study, with these two groups emphasising VA and the negative impact perception.^19^

Improving patient QoL and implementing quality improvement programs are essential in HD patients. Assessing satisfaction, daily life impact of the VA and physical and psychological discomfort can trigger the implementation of interventions to reduce the burden of disease.

The study results represent a higher impact in future research with the application of this tool as a screening method for assessing HD patients regarding their vascular access. Since the patient-centred approach implementation in recent years, the need to individualise the VA decision needs to evaluate the patient, their needs, and their condition, with a capacity to determine the proper access regarding the patient condition and their satisfaction levels with an appraisal for their QoL.

This study had limitations: the relatively small sample size and the convenience of sample recruiting. Also, the patient’s characteristics may influence the satisfaction levels regarding the VA, and further studies are needed to assess more factors that can affect VA satisfaction levels.

## Conclusion

The SF-VAQ is a robust instrument with excellent psychometric properties. Even with the limited sample used in this study and convenience selection methodologies as a limitation, the reliability and validity of this scale are meaningful because of the first time reported Portuguese version.
